# Post-mortem imaging in suspected child physical abuse: a systematic review

**DOI:** 10.1007/s00330-025-12172-1

**Published:** 2026-01-23

**Authors:** Brendan S. Kelly, Rick R. van Rijn, Harry Bliss, Timothy Cain, Jamie Carter, Heather Chesters, Judith Fronczek, Amit Haboosheh, Elaine Kan, Ola Kvist, Fox Marttinen, Michelle Nagtegaal, Padma Rao, Claire Robinson, Jai Sidpra, Amaka C. Offiah, Owen J. Arthurs

**Affiliations:** 1 Department of Radiology, Great Ormond Street Hospital for Children NHS Foundation Trust, London, UK; 2 Insight Centre for Data Analytics, University College Dublin, Dublin, Ireland; 3 Department of Radiology and Nuclear Medicine, University of Amsterdam, Amsterdam UMC, Amsterdam, The Netherlands; 4 Learning Academy, Great Ormond Street Hospital for Children, London, UK; 5 The Royal Children’s Hospital Melbourne, Melbourne, VIC Australia; 6 Consultant Paediatrician, Designated Doctor for Safeguarding Children (Brighton & Hove & West Sussex) & Designated Paediatrician for Child Deaths, Surrey, UK; 7 Honorary Clinical Senior Lecturer & Deputy Lead Module 101, Brighton and Sussex Medical School, Brighton, UK; 8 UCL Great Ormond Street Institute of Child Health Library, University College London, London, UK; 9 Forensic pathologist, Victorian Institute of Forensic Medicine, Department of Pathology, Southbank, VIC Australia; 10 Adjunct Lecturer, Department of Forensic Medicine, Monash University, Clayton, VIC Australia; 11 Attending Neuroradiologist, Head of Fetal MRI, and adult head and neck imaging Division of Neuroradiology Department of Radiology Hadassah Ein Karem Hospital, Jerusalem, Israel; 12 Department of Radiology, Hong Kong Children’s Hospital, KLN, Hong Kong; 13 Department of Radiology, Columbia University Medical Center, New York, NY USA; 14 Department of Women’s and Children’s Health, Karolinska Institute, Stockholm, Sweden; 15 HUS Diagnostic Center, Radiology, South Karelia Central Hospital, Lappeenranta, Finland; 16 Department of Forensic Medicine, Netherlands Forensic Institute, The Hague, The Netherlands; 17 Imaging Department, Leicester Royal Infirmary, University Hospitals of Leicester NHS Trust, Leicester, UK; 18 Developmental Biology & Cancer Section, University College London Great Ormond Street Institute of Child Health, London, UK; 19 Chair of Paediatric Musculoskeletal Imaging and Honorary Consultant Paediatric Radiologist, Division of Clinical Medicine, University of Sheffield, Sheffield, UK; 20 Sheffield Children’s NHS Foundation Trust, Sheffield, UK

**Keywords:** Forensic imaging, Paediatric radiology, Radiography, Magnetic resonance imaging, Computed tomography

## Abstract

**Objectives:**

As post-mortem (PM) imaging in children becomes more common, there is a need to review the available evidence for its diagnostic yield in suspected child physical abuse. The aim of this review is to synthesise current evidence, assess study quality, and identify ongoing challenges.

**Materials and methods:**

Following PRISMA guidelines, databases were searched until 31 December 2024. Original research articles reporting data on at least ten children with PM imaging in the context of physical abuse were included. Titles and abstracts were screened by two expert reviewers; full texts were assessed by a third, independent reviewer and one of the previous reviewers. Data was extracted by one of 12 experts and independently verified. The study risk of bias was evaluated with the ROBINS-I tool. Study heterogeneity precluded meta-analysis, resulting in descriptive synthesis.

**Results:**

Eighteen out of 1687 potential papers were included. Seven described PM radiography, five post-mortem computed tomography (PMCT), four both PM radiography plus PMCT, and two post-mortem magnetic resonance imaging (PMMR). All but one were retrospective, and most (11/18, 61%) had a moderate-to-high risk of bias. Post-mortem skeletal survey (PMSS) detected subtle fractures, particularly corner metaphyseal fractures. PMCT provided a high-resolution assessment of injuries, particularly rib fractures. PMMR contributed soft-tissue and intracranial detail. All studies emphasised the importance of correlating autopsy findings. Technical variation and potential biases limited direct comparisons between studies.

**Conclusion:**

PM imaging can reveal important injury patterns that may be overlooked by autopsy. Nevertheless, standardised imaging methods and larger prospective trials are needed to reduce bias and establish best-practice guidelines.

**Key Points:**

***Question*** *What is the evidence for PM radiologic imaging in suspected physical abuse of children?*

***Findings**** PM imaging complements autopsy, but diagnostic accuracy varies by modality. Study heterogeneity and bias limit current evidence*.

***Clinical relevance**** PM imaging can detect injuries missed at autopsy in child abuse cases. Standardised protocols and higher-quality studies are urgently needed*.

**Graphical Abstract:**

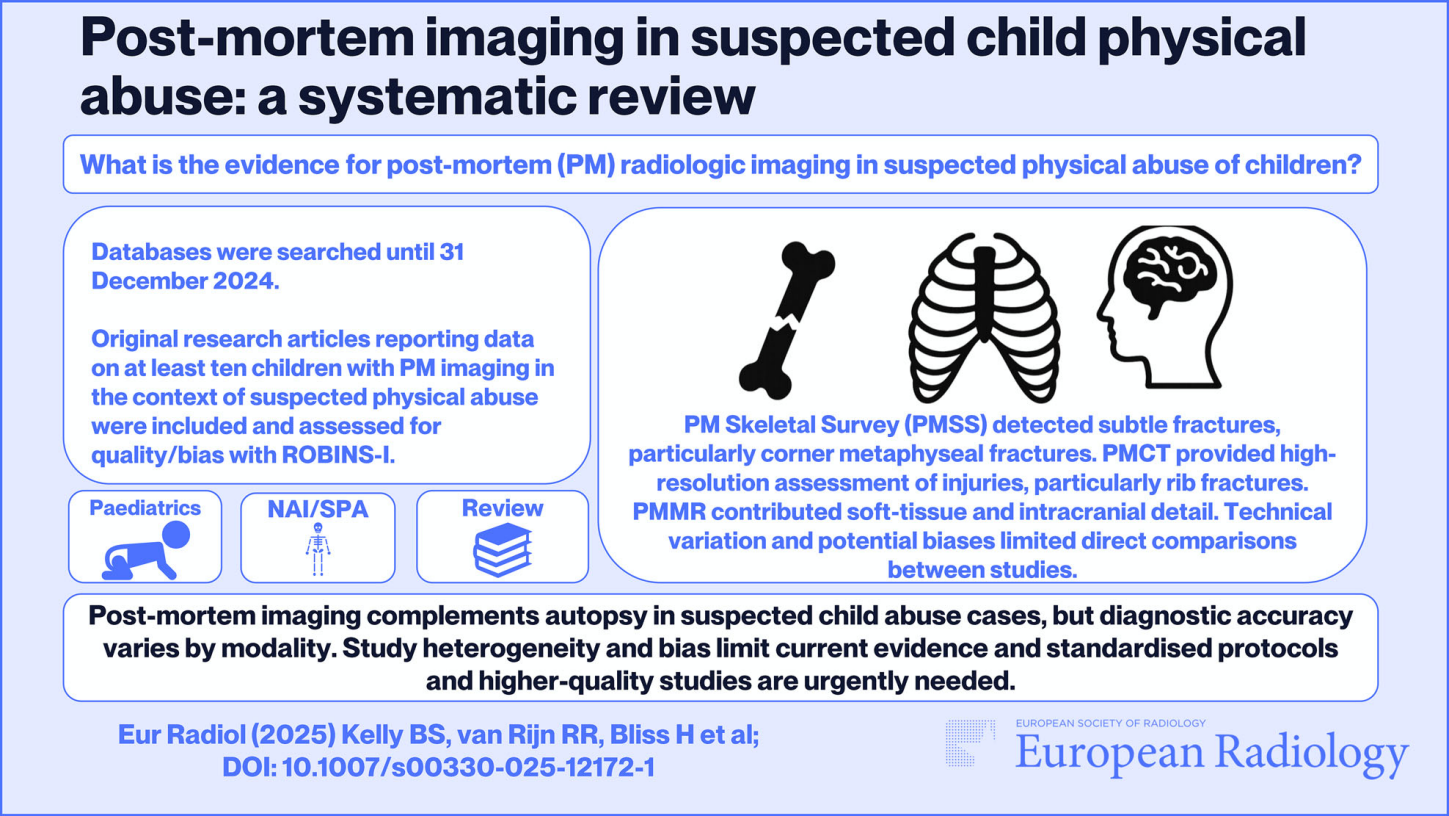

## Introduction

Physical abuse of children is a global problem that causes significant morbidity and mortality [1]. Most abuse happens in infants and young children (aged < 2 years), who are 120 times more likely to suffer physical abuse than children over the age of five years [2]. Determining whether inflicted injuries contributed to or caused a child’s death carries significant clinical and legal implications, making this a critical concern for both healthcare providers and forensic teams [3, 4].

Autopsy remains the gold standard for identifying the cause and manner of death in children with suspected physical abuse [5]. However, even when an autopsy is mandated in forensic cases, it does not always yield a definitive cause of death [6]. While practices vary between countries, further workup is often not initiated if no cause of death relevant to legal proceedings is found [7]. This contrasts with the clinical setting, where the cause of death is the main reason for performing a post-mortem evaluation. Post-mortem (PM) imaging has emerged as an adjunct to help clarify injury patterns, detect occult findings that may not be discovered during an autopsy (e.g. subtle fractures), and to assist the forensic pathologist in performing the autopsy [8]. It has seen varying adoption across countries, even within Europe, for example, post-mortem computed tomography (PMCT) is unused in some jurisdictions but is clearly recommended in Germany [9].

This area of practice poses challenges for the general radiologist, given the importance of neither over- nor under-diagnosing abnormalities, as well as perceived high litigation rates [1]. Up-to-date evidence and guidelines are therefore crucial to ensure uniformly high-quality care based on the best available data, enabling more confident and competent clinical practice [3]. In particular, recent guidelines for the imaging of suspected child physical abuse have lacked clarity and completeness with respect to PM imaging [10, 11]. Furthermore, despite growing interest in PM imaging, the evidence supporting its use in suspected child abuse cases remains inconsistent [12, 13]. There is considerable variation in practice and heterogeneous evidence concerning imaging protocols and interpretation [8, 14, 15]. Although multiple PM imaging techniques, including PMCT, micro CT, post-mortem magnetic resonance imaging (PMMR), and post-mortem ultrasound (PMUS) have been explored, there is little consensus on which modality yields the most accurate or practical information, particularly in infants and children [6, 12]. The lack of formal guidelines or standardised protocols further complicates adoption by radiologists and clinicians [1, 6, 16].

Our aim was to investigate the evidence for PM radiologic imaging in suspected physical abuse of children. To this end, the current systematic review aims to synthesise current literature on PM imaging in suspected physical abuse in children and consider its diagnostic performance. Following the preferred reporting items for systematic reviews and meta-analyses (PRISMA) guidelines [17], relevant studies are identified, appraised, and summarised to consider the relative performance, advantages and limitations of these imaging techniques. A bias assessment is performed using risk of bias in non-randomised studies of interventions (ROBINS-I) [18] to ensure study methodological rigour and transparency. The findings may guide future clinical practice, inform policy recommendations, and address the urgent need for reliable, consistent methods of PM evaluation in suspected child abuse.

## Materials and methods

This study was undertaken by the ‘International Guidelines for the Imaging Investigation of Suspected Child Physical Abuse’ (IGISPA) Post-Mortem Imaging Subgroup in accordance with PRISMA [17] guidelines (Supplementary Material 1). PRISMA was used as they are the internationally agreed standard guidelines for systematic reviews. The systematic review protocol was recorded in the minutes of a regular periodic IGISPA Post-Mortem Imaging Subgroup meeting, but it was not prospectively published. As a systematic review of published data, institutional review board approval and informed consent were not required. A population, intervention, comparator, outcome (PICO) based approach was used to define study inclusion and exclusion criteria. The target population was defined as neonates, infants, children, and adolescents—excluding fetuses, adults, and non-human remains. Acceptable interventions/exposures/tests were PMUS, PM radiography, PMCT, PMMR, and micro-CT. Comparators or contextual assessments included the same imaging modalities, autopsy, and physical examination. Sensitivity and specificity were the primary outcomes of interest (with autopsy as the reference standard); however, heterogeneous reporting of results was expected.

Randomised controlled trials (RCTs), observational cohort studies, and case series with ten or more confirmed abuse cases were included—excluding review articles, case reports, case series reporting data on fewer than ten patients, pictorial essays, letters to the editor, commentaries, and clinical vignettes. All foetal imaging papers were excluded. Also excluded were any manuscripts that addressed perinatal or neonatal imaging outside of the context of suspected physical abuse. Please see Table 1. The references of included papers and relevant review papers were also searched for potentially relevant articles.

**Table 1:** Inclusion and exclusion criteria

Inclusion criteria	Exclusion criteria
Original research studies published on or before 31 December 2024	Review articles, case reports, pictorial essays, letters, commentaries, clinical vignettes
RCTs, observational cohort studies, or case series reporting ≥ 10 cases	Case series reporting < 10 cases
Population: neonates, infants, children, and adolescents	Fetuses, adults, or non-human remains
Clinical context: suspected child physical abuse	Studies not addressing physical abuse (e.g. perinatal death without suspected abuse)
Imaging modality: PMUS, post-mortem radiography, post-mortem CT (PMCT), PMMR, and micro-CT	Studies not involving post-mortem imaging
Comparator: autopsy, physical examination, or other PM imaging	Not applicable
Outcomes of interest: diagnostic sensitivity and/or specificity (with autopsy as the reference standard)	Studies not reporting relevant diagnostic data or outcomes
No restriction on publication language or status	—

Summary of inclusion and exclusion criteria applied in this systematic review. Eligible studies were those reporting original research on PM imaging in the context of suspected child physical abuse

Original research articles published before 31 December 2024 were retrospectively retrieved and analysed. All included databases were searched from their inception. All eligible articles were considered for inclusion, regardless of publication language or status. The databases Medline (Ovid), Embase (Ovid), CINAHL Plus (EBSCOhost), and Web of Science Core Collection were searched for relevant publications. Our full search strategy has been included as a supplement (Supplementary Material 2).

Two expert reviewers (O.A. and R.R.v.R.) independently screened all retrieved titles and abstracts against the a priori defined study inclusion criteria; any article meeting these criteria or lacking sufficient information to determine this was retrieved in full. O.A. and R.R.v.R. are both professors of radiology with a special interest in PM imaging and 13 and 22 years of experience, respectively. Full-text screening was performed by O.A. and B.K. (a radiologist with 2 years of post-fellowship examination experience, undertaking subspecialisation training in paediatric radiology and PM imaging). Discrepancies in study inclusion decisions were resolved in consensus between B.K. and O.A.

The included papers were circulated by B.K., one of the 12 members of the IGISPA Post-Mortem Imaging Subgroup. Articles were distributed in this manner to avoid potential conflicts of interest and to ensure that two experts (B.K. and an IGISPA panel member) assessed each paper independently. Disagreements were resolved by consensus discussion with O.A. and/or R.R.v.R. To ensure consistency, a data extraction tool was circulated to members and is available as a supplement. Data extracted included study design, sample size, patient demographics, imaging modality, and bias assessment data. Risk of bias was evaluated using the ROBINS-I tool [18]. This tool was chosen as the most applicable Cochrane tool available at the time of data extraction, as the use of RoB 2.0 is restricted to randomised trials and would not be applicable in this context.

Due to the heterogeneity of study methodologies and imaging modalities, a pooled meta-analysis was not performed, nor were effect measures calculated. Instead, descriptive synthesis was undertaken and results tabulated to compare study characteristics and outcomes. Quality of evidence was appraised qualitatively, given the primarily descriptive or comparative nature of the studies. No additional statistical methods were used beyond descriptive summaries.

## Results

**Fig. 1:**
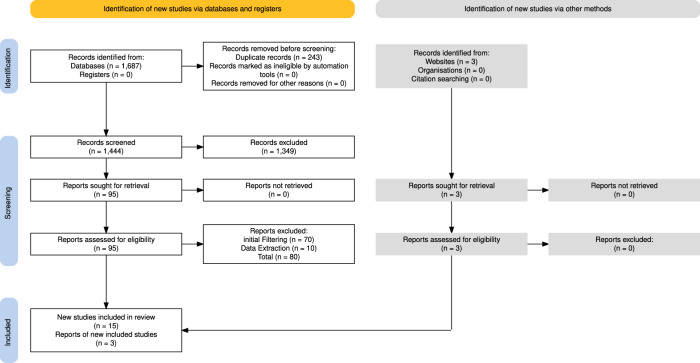
PRISMA Flowchart of study inclusion showing initial retrieval (*n* = 1444), after screening (*n* = 756), full-text review (*n* = 95), additional references identified (*n* = 3), and final inclusion (*n* = 18)

A total of 1687 papers, of which 1444 were unique, were identified through the initial database search. After title and abstract screening, 95 articles underwent full-text review. Three additional records were found by reviewing reference lists. Ultimately, 18 studies met all criteria and were included in the final analysis (Fig. 1).

Of the 18 included publications, seven investigated skeletal survey/radiography [19–25], five focused on PMCT [26–30], four reported data on both skeletal survey and PMCT [31–34], and two assessed PMMR [35, 36]. Only one study was prospective [36]. Ages of included subjects ranged from 3 days to 17 years. Table 2 summarises the main characteristics of included studies. Heterogeneity was observed in study design, imaging protocols, and reporting methods, preventing meaningful quantitative synthesis or meta-analysis.

**Table 2:** Summary of included studies, showing study design (prospective or retrospective), sample size, patient age ranges, imaging modality/modalities used, and key outcome measures

Author	Year	Title	Country	Single-centre/multi-centre	*N*	Age range	Modality
Kleinman [22]	1995	Inflicted skeletal injury: a postmortem radiologic-histopathologic study in 31 infants	USA	Single-centre	31	3 weeks–11 months	PM radiography
Kleinman [23]	1995	Relationship of the subperiosteal bone collar to metaphyseal lesions in abused infants	USA	Single-centre	10	1–5 months	PM radiography
Blaine [35]	1996	Postmortem cranial MRI and autopsy correlation in suspected child abuse	USA	Single-centre	11	3– 26 months	PMMR
McGraw [24]	2001	Postmortem radiography after unexpected death in neonates, infants, and children: Should imaging be routine?	USA	Multicentre	14	0–2years	PM radiography
deLange [19]	2007	[24] Death in infants and children compared to autopsy	Norway	Multicentre	110	0–3 years	PM radiography
Hong [31]	2011	Value of postmortem thoracic CT over radiography in imaging of paediatric rib fractures	Ca	Single-centre	56	8 days–8 years	PM radiography, PMCT
Arthurs [25]	2012	PM skeletal surveys in suspected non-accidental injury	UK	Single-centre	195	2 days–5 years	PM radiography
Proisy [32]	2013	Whole-body PMCT compared with autopsy in the investigation of unexpected death in infants and children	France	Single-centre	44	0–8 years	PM radiography, PMCT
Sieswerda-Hoogendoorn [30]	2013	The value of postmortem CT in neonaticide in case of severe decomposition: description of 12 cases	Ned	Single-centre	12	< 12 months	PMCT
Thayyil [36]	2013	PM MRI vs conventional autopsy in fetuses and children: a prospective validation study	UK	Multicentre	15	0–16 years	PMMR
Sieswerda-Hoogendoorn [29]	2014	Postmortem CT compared to autopsy in children; concordance in a forensic setting	Ned	Single-centre	71	3 months–6 years	PMCT
Arthurs [26]	2015	Ventilated PMCT in children: feasibility and initial experience	UK	Single-centre	12	3–304 days	PMCT
Rowbotham [33]	2021	An evaluation of the differences in paediatric skeletal trauma between fatal simple short falls and physical abuse blunt impact loads: an international multicentre pilot study	Multi	Multicentre	21	6 days–9 years	PM radiography, PMCT
Speelman [28]	2022	PMCT plus forensic autopsy for determining the cause of death in child fatalities	SA/UK	Multicentre	15	0–17 years	PMCT
Wessels [21]	2022	Fatal non-accidental injury in South Africa: a Gauteng hospital’s perspective on the incidence and fracture types in PM skeletal surveys	SA	Single-centre	73	0–13 years	PM radiography
Henry [20]	2023	Yield of Postmortem Skeletal Surveys in Infants Presenting to Emergency Care With Sudden and Unexpected Death	USA	Single-centre	73	0–10 months	PM radiography
Lathrop [27]	2023	Can computed tomography replace or supplement autopsy?	USA	Single-centre	12	0–10 years	PMCT
Shelmerdine [34]	2024	Post-mortem skeletal survey (PMSS) versus PMCT for the detection of corner metaphyseal lesions (CML) in children	UK	Single-centre	10	8 days–9 months	PM radiography, PMCT

Of PM radiography papers, a majority (5/7 [19–25], 71%) compare radiographic findings with autopsy results. Across these, PM radiography detects fractures that are frequently missed by standard autopsy, especially in small or decomposed children. For instance, a South African cohort [21], with a median age of 28 months, found that limb fractures, often difficult to appreciate on gross examination, were readily identifiable on PM radiography. Furthermore, the specific value of PM radiography for the detection of metaphyseal fractures, typical of inflicted trauma, the “corner” or “bucket-handle” appearances, has been described [23].

Although few studies report formal test performance metrics, a common estimate for the sensitivity for fracture detection of PM skeletal survey is approximately 50–60% [22], when compared to combined skeletal survey and autopsy or cross-sectional imaging. Regarding the value of specimen radiography, two studies [22, 23] revealed that targeted specimen radiography and histological sectioning improve fracture detection and characterisation.

One paper focusing on infants presenting to the emergency department [20] reported high radiologist interobserver agreement (Kappa [k] = 0.85) for confirming definite fractures, yet poor agreement for “possible” fractures (k = −0.01), suggesting that subtle or equivocal radiographic findings remain difficult to interpret. This underscores the need for specialised paediatric radiography and radiology expertise and the potential added value of double reporting where available.

PMCT studies had a wide variety of research questions, from focusing on ventilation [26], to their use in severe decomposition [30], and comparison with autopsy [28, 29]. Good sensitivity of 70–84% for the cause of death was demonstrated when compared with autopsy, though specificity was much lower (30%) when reported [29]. Of the four studies comparing PMCT to PM radiography [30, 32–34], PMCT was markedly more sensitive than radiography, with sensitivity estimates of 51–85% for PMCT vs 29–46% for radiographs in detecting rib fractures. This underscores PMCT’s superiority for thoracic osseous detail [31]. In one study, while PMCT was reported to have slightly higher specificity (92.7% vs 90.5%), skeletal survey was reported to have higher sensitivity (69.6% vs 60.5%). In this context, corner metaphyseal fractures (CMFs) may be better visualised on carefully positioned, high-detail plain radiographs [34].

One brain [35] and one whole-body [36] PMMR paper were included. For brain PMMR, evidence of significant head injury was found in eight of the 11 included children by both PMMR and autopsy; three showed no head injury on either modality [35]. PMMR was superior in demonstrating mastoid fluid, focal axonal shearing, cortical abnormalities, and ischaemic changes, while autopsy better identified subarachnoid haemorrhage and suture separation. In a prospective study [36], whole-body PMMR achieved 89% concordance with full autopsy for determining the cause of death or major pathological lesions that either contributed to, or were responsible for, death. Accuracy was highest in younger children and declined in older children.

**Fig. 2:**
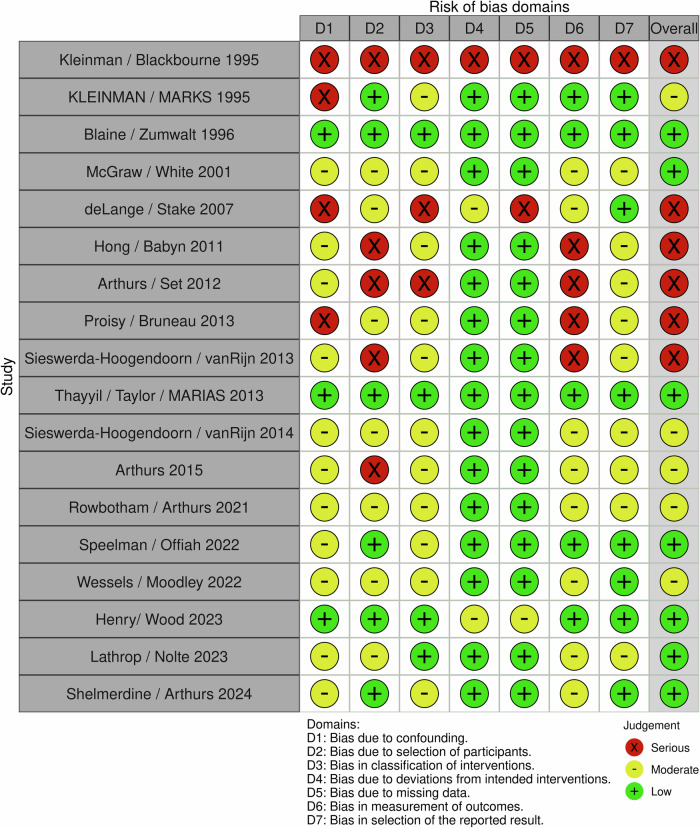
ROBINS-I bias assessment of included studies

Risk of bias was assessed across seven domains according to the ROBINS-I tool, shown in Fig. 2. Of the 18 included studies, 7 (39%) were judged to be at low overall risk of bias, 5 (27%) at moderate risk, and 6 (33%) at serious risk. Regarding the different risk of bias Domains (D), bias due to confounding (D1) was a common area of concern, with only three studies (17%) rated low risk. Eleven studies (61%) were assessed as having moderate risk, and four (22%) were rated as serious risk due to lack of adjustment for key covariates (e.g. age, cause of death) or inadequate reporting of potential confounders.

Bias due to the selection of participants (D2) varied substantially. Six studies (33%) demonstrated low risk, often due to clear inclusion criteria and recruitment strategies. However, five studies (28%) were judged as serious risk, largely due to unclear or selective inclusion of PM cases. The remaining seven studies (39%) were considered at moderate risk. Classification of interventions (D3) was similarly varied, with four studies (22%) rated low risk, particularly those with consistent imaging protocols and blinding of assessors. Only three studies (17%) were rated serious due to vague or retrospective assignment of imaging modalities. Eleven studies (61%) were rated as moderate risk. Bias due to deviations from intended interventions (D4) and missing data (D5) were more robust, with 15/18 (83%) achieving a low risk of bias, likely related to how the imaging modality was often the intervention being studied. Measurement bias (D6) was more heterogeneous, with five studies (28%) at either serious or low risk. The remaining eight studies (44%) were rated as moderate risk. Selective reporting (D7) was a concern in only one study (6%), with the remaining 17 (94%) at moderate or high.

One older study [22] was rated as being at serious risk of bias across all domains, and one study [36], the only prospective study included, was rated at low risk in all domains. Importantly, all six studies with an overall serious risk of bias were published in or before 2013, highlighting an improvement in methodological rigour in more recent literature. Notably, all studies published from 2023 onward were rated as low overall risk, reflecting increasing standardisation of imaging protocols and reporting practices.

## Discussion

The primary aim of this systematic review was to review the available literature in PM radiologic imaging of suspected child physical abuse. We also sought to assess its diagnostic performance, advantages and limitations. The findings indicate a dearth of literature underlying the use of radiologic imaging SPA. The available literature shows that PM radiography, PMCT, and PMMR each offer unique benefits, often complementing autopsy by detecting fractures or soft-tissue injuries that dissection alone can miss. Skeletal surveys were shown to be more sensitive for uncovering occult bony injuries, particularly in infants with CMFs [34], while PMCT and PMMR helped to delineate complex fracture patterns, soft-tissue pathology, and intracranial lesions [27, 35, 36]. All papers emphasised that autopsy, often aided by targeted histopathology, is indispensable for definitively confirming fractures and ruling out artefacts. PM imaging prior to autopsy can direct pathologists to suspicious sites for closer inspection. Nonetheless, significant methodological heterogeneity and the high risk of bias identified in many publications restricted our ability to make definitive statements about the accuracy of any single imaging modality. Indeed, the heterogeneity of the studies precluded formal meta-analysis or a detailed comparative assessment of diagnostic accuracy.

When comparing these results with the existing literature, the evidence consistently supports integrating imaging into forensic investigations of suspected child physical abuse [3, 6]. Previous reports have shown that post-mortem skeletal survey (PMSS) can detect fractures at different healing stages, raising the clinical suspicion for recurrent trauma [1]. The reviewed studies reinforce this conclusion, highlighting CMFs and other high-specificity fracture patterns that suggest inflicted harm may be better seen on PMSS [34]. PMCT has been noted to excel in detecting rib and vertebral injuries, mirroring findings from adult PM imaging research, while PMMR has received attention for identifying more subtle soft-tissue and intracranial pathologies [35, 36]. Collectively, these modalities can guide autopsy, improve diagnostic confidence, and potentially strengthen the medicolegal process [1]

The heterogeneity in risk of bias across included studies has direct implications for interpreting the strength of the current evidence base in PM imaging for suspected child abuse. Confounding was a particularly persistent issue, with only 17% of studies at low risk, reflecting the lack of adjustment for key variables such as cause of death, age, or clinical context. This undermines confidence in reported associations between imaging findings and abuse-related injuries. Similarly, selection bias remained problematic in over half of the studies, often due to retrospective inclusion or unclear case selection criteria, which could skew both prevalence estimates and diagnostic accuracy outcomes. These biases are not theoretical concerns; rather, they affect the credibility of conclusions drawn from older studies.

Several biases and limitations emerged across this body of work. Many studies were retrospective, used inconsistent imaging protocols, or had variably experienced readers, limiting direct comparability. In the retrospective setting, readers' knowledge of abuse suspicions could have introduced bias, as readers might scrutinise images more closely for cases in which abuse was expected. Sample sizes were often small, and detailed clinical or forensic follow-up data were frequently absent. The interval between death and imaging also varied considerably and may have influenced image quality, particularly in cases where decomposition had progressed. Addressing these limitations will require larger prospective studies using standardised imaging protocols, blinded interpretations, and consistent timing. Incorporating structured radiological assessment of fracture age, use of advanced imaging systems, and histopathological correlation would further enhance methodological robustness.

Encouragingly, an improvement in methodological rigour was observed in more recent publications. All studies rated as having serious overall risk were published in or before 2013, whereas all studies from 2023 onward were rated low risk. This temporal shift likely reflects the increasing use of standardised imaging protocols, clearer inclusion criteria, and prospective design principles. Notably, the only prospective study included in this review [36] was rated as low risk across all domains, demonstrating the feasibility and value of rigorous design even in a challenging forensic context. These findings suggest that future guidelines and medico-legal interpretations should prioritise high-quality, recent evidence and reinforce the need for prospective, standardised, and multi-disciplinary studies to drive the field forward.

Furthermore, we acknowledge that by formulating our research question to focus on postmortem imaging, thereby including only postmortem cases with confirmed physical abuse and excluding abuse cases in living children, there is a potential selection bias. Future research could choose to widen this literature search to include imaging in living children with suspected physical abuse.

In conclusion, PM imaging can substantially improve the detection and characterisation of injuries in suspected child physical abuse, especially when integrated with conventional autopsy. This review highlights the importance of radiography for identifying subtle bony lesions, the utility of PMCT for assessing complex skeletal structures, and the added value of PMMR for delineating soft-tissue and intracranial pathologies. Although variations in study methodology and inherent biases limit definitive conclusions, these findings support a multi-modality imaging approach in forensic paediatric practice. Future studies featuring standardised imaging protocols, careful blinding, and robust outcome measures are warranted to strengthen the evidence base and offer clearer guidance for clinicians and other healthcare and medicolegal professionals.

## Supplementary information

ELECTRONIC SUPPLEMENTARY MATERIAL

## Abbreviations

CT: Computed tomography
IGISPA: International guidelines for the investigation of suspected child physical abuse
MRI: Magnetic resonance imaging
PM: Post-mortem
PMCT: Post-mortem computed tomography
PMMR: Post-mortem magnetic resonance imaging
PMSS: Post-mortem skeletal survey
PMUS: Post-mortem ultrasound
PRISMA: Preferred reporting items for systematic reviews and meta-analyses
RCT: Randomised controlled trial
ROBINS-I: Risk of bias in non-randomised studies of interventions
