# Supplementary material for: Post-mortem imaging in suspected child physical abuse: a systematic review
Source: Eur Radiol. 2026 Jan 23;36(6):4739–48. doi: 10.1007/s00330-025-12172-1 (PMC13212398; doi:10.1007/s00330-025-12172-1)
Supplement: Supplementary file 1 — ELECTRONIC SUPPLEMENTARY MATERIAL [file 330_2025_12172_MOESM1_ESM.pdf]

# Post-mortem imaging in suspected child physical abuse: a systematic review

## ELECTRONIC SUPPLEMENTARY MATERIAL

Eur Radiol (2025) Kelly BS, van Rijn RR, Bliss H, et al.

| Section and Topic | Item # | Checklist item | Location where item is reported |
|---|---|---|---|
| **TITLE** | | | |
| Title | 1 | Identify the report as a systematic review. | Title |
| **ABSTRACT** | | | |
| Abstract | 2 | See the PRISMA 2020 for Abstracts checklist. | Y |
| **INTRODUCTION** | | | |
| Rationale | 3 | Describe the rationale for the review in the context of existing knowledge. | Intro p 3 |
| Objectives | 4 | Provide an explicit statement of the objective(s) or question(s) the review addresses. | Intro p4 |
| **METHODS** | | | |
| Eligibility criteria | 5 | Specify the inclusion and exclusion criteria for the review and how studies were grouped for the syntheses. | MM p1 |
| Information sources | 6 | Specify all databases, registers, websites, organisations, reference lists and other sources searched or consulted to identify studies. Specify the date when each source was last searched or consulted. | MM p2 |
| Search strategy | 7 | Present the full search strategies for all databases, registers and websites, including any filters and limits used. | MM p 3 |
| Selection process | 8 | Specify the methods used to decide whether a study met the inclusion criteria of the review, including how many reviewers screened each record and each report retrieved, whether they worked independently, and if applicable, details of automation tools used in the process. | MM p 4 |
| Data collection process | 9 | Specify the methods used to collect data from reports, including how many reviewers collected data from each report, whether they worked independently, any processes for obtaining or confirming data from study investigators, and if applicable, details of automation tools used in the process. | MM p 5 |
| Data items | 10a | List and define all outcomes for which data were sought. Specify whether all results that were compatible with each outcome domain in each study were sought (e.g. for all measures, time points, analyses), and if not, the methods used to decide which results to collect. | MM p 5 |
| | 10b | List and define all other variables for which data were sought (e.g. participant and intervention characteristics, funding sources). Describe any assumptions made about any missing or unclear information. | MM p 5 |
| Study risk of bias assessment | 11 | Specify the methods used to assess risk of bias in the included studies, including details of the tool(s) used, how many reviewers assessed each study and whether they worked independently, and if applicable, details of automation tools used in the process. | MM p 6 |
| Effect measures | 12 | Specify for each outcome the effect measure(s) (e.g. risk ratio, mean difference) used in the synthesis or presentation of results. | MM p 6 |
| Synthesis methods | 13a | Describe the processes used to decide which studies were eligible for each synthesis (e.g. tabulating the study intervention characteristics and comparing against the planned groups for each synthesis (item #5)). | NA |
| | 13b | Describe any methods required to prepare the data for presentation or synthesis, such as handling of missing summary statistics, or data conversions. | NA |
| | 13c | Describe any methods used to tabulate or visually display results of individual studies and syntheses. | NA |
| | 13d | Describe any methods used to synthesize results and provide a rationale for the choice(s). If meta-analysis was performed, | NA |

| Section and Topic | Item # | Checklist item | Location where item is reported |
|---|---|---|---|
| | | describe the model(s), method(s) to identify the presence and extent of statistical heterogeneity, and software package(s) used. | |
| | 13e | Describe any methods used to explore possible causes of heterogeneity among study results (e.g. subgroup analysis, meta-regression). | NA |
| | 13f | Describe any sensitivity analyses conducted to assess robustness of the synthesized results. | NA |
| Reporting bias assessment | 14 | Describe any methods used to assess risk of bias due to missing results in a synthesis (arising from reporting biases). | MM P 6 |
| Certainty assessment | 15 | Describe any methods used to assess certainty (or confidence) in the body of evidence for an outcome. | NA |
| **RESULTS** | | | |
| Study selection | 16a | Describe the results of the search and selection process, from the number of records identified in the search to the number of studies included in the review, ideally using a flow diagram. | R P 1 |
| | 16b | Cite studies that might appear to meet the inclusion criteria, but which were excluded, and explain why they were excluded. | R P 1 |
| Study characteristics | 17 | Cite each included study and present its characteristics. | R P 2 |
| Risk of bias in studies | 18 | Present assessments of risk of bias for each included study. | R P 9 |
| Results of individual studies | 19 | For all outcomes, present, for each study: (a) summary statistics for each group (where appropriate) and (b) an effect estimate and its precision (e.g. confidence/credible interval), ideally using structured tables or plots. | TAB 1 |
| Results of syntheses | 20a | For each synthesis, briefly summarise the characteristics and risk of bias among contributing studies. | NA |
| | 20b | Present results of all statistical syntheses conducted. If meta-analysis was done, present for each the summary estimate and its precision (e.g. confidence/credible interval) and measures of statistical heterogeneity. If comparing groups, describe the direction of the effect. | NA |
| | 20c | Present results of all investigations of possible causes of heterogeneity among study results. | FIG 1 |
| | 20d | Present results of all sensitivity analyses conducted to assess the robustness of the synthesized results. | NA |
| Reporting biases | 21 | Present assessments of risk of bias due to missing results (arising from reporting biases) for each synthesis assessed. | FIG 2 |
| Certainty of evidence | 22 | Present assessments of certainty (or confidence) in the body of evidence for each outcome assessed. | NA |
| **DISCUSSION** | | | |
| Discussion | 23a | Provide a general interpretation of the results in the context of other evidence. | D P 1 |
| | 23b | Discuss any limitations of the evidence included in the review. | D P 2 |
| | 23c | Discuss any limitations of the review processes used. | D P 3 |
| | 23d | Discuss implications of the results for practice, policy, and future research. | D P 4 |
| **OTHER INFORMATION** | | | |
| Registration and protocol | 24a | Provide registration information for the review, including register name and registration number, or state that the review was not registered. | Mm p 1 |
| | 24b | Indicate where the review protocol can be accessed, or state that a protocol was not prepared. | Na |

| Section and Topic | Item # | Checklist item | Location where item is reported |
|---|---|---|---|
| | 24c | Describe and explain any amendments to information provided at registration or in the protocol. | Na |
| Support | 25 | Describe sources of financial or non-financial support for the review, and the role of the funders or sponsors in the review. | DISC |
| Competing interests | 26 | Declare any competing interests of review authors. | DISC |
| Availability of data, code and other materials | 27 | Report which of the following are publicly available and where they can be found: template data collection forms; data extracted from included studies; data used for all analyses; analytic code; any other materials used in the review. | DISC |

**Ovid MEDLINE(R) ALL <1946 to July 12, 2024>**
**Search run: 15/07/2024 updated 31/12/2024**

1 exp Child Abuse/ 35639
2 Physical Abuse/ 1249
3 Battered Child Syndrome/ 667
4 Shaken Baby Syndrome/ 682
5 Forensic Imaging/ 1
6 Forensic Medicine/ 22725
7 (AHT or NAI or NAHI or IHT or SBS).ti,ab,kf. 13943
8 (abus* adj5 (physical or suspect* or child* or infant* or injur* or head trauma)).ti,ab,kf. 36742
9 ((inflicted or nonaccidental or non-accidental) adj3 (injur* or trauma or fracture*)).ti,ab,kf. 4489
10 (maltreat* or physical violence or physical punishment or short fall).ti,ab,kf. 15743
11 (child* abuse or child* neglect or battered child* or parent-infant traumatic stress syndrome or shaken baby syndrome or shaken impact syndrome or whiplash-shake injury syndrome).ti,ab,kf. 16567
12 forensic*.ti,ab,kf. 58860
13 or/1-12 145202
14 Child, Preschool/ or exp infant/ 1757852
15 exp Pediatrics/ 63773
16 (child* or baby or babies or newborn* or new-born* or neonat* or neo-nat* or preterm* or pre-term* or prematur* or pre-matur* or infant* or toddler* or preschool* or pre-school* or kid or kids or boy* or girl* or pediatric* or paediatric* or under 5* or under five* or under two* or under 2* or under one* or under 1* or shaken impact syndrome or whiplash-shake injury syndrome or SBS).ti,ab,kf. 2912676
17 or/14-16 3485526
18 Diagnostic Imaging/ 47072
19 diagnostic imaging.fs. 1508156
20 (image* or imaging or MRI or CT or computed tomography or neuroimaging or radiolog* or radiograph* or bone survey* or skeletal survey* or bone scintigraphy or virt?ops*).ti,ab,kf. 2500362
21 or/18-20 3285815
22 Autopsy/ or Postmortem Imaging/ 45908
23 (autop* or preautop* or necrops* or necroscop* or obducti* or postmort* or post mort* or virt?ops*).ti,ab,kf. 274018
24 or/22-23 293720
25 13 and 17 and 21 and 24 387

**Embase Classic+Embase(Ovid) <1947 to 2024 July 12>**
**Search run: 15/07/2024**

1 exp child abuse/ 47654
2 physical abuse/ 9239
3 battered child syndrome/ 1291
4 shaken baby syndrome/ 1339
5 forensic medicine/ 37487
6 (AHT or NAI or NAHI or IHT or SBS).ti,ab,kf. 15893

7	(abus* adj5 (physical or suspect* or child* or infant* or injur* or head trauma)).ti,ab,kf.	46928
8	((inflicted or nonaccidental or non-accidental) adj3 (injur* or trauma or fracture*)).ti,ab,kf.	6216
9	(maltreat* or physical violence or physical punishment or short fall).ti,ab,kf.	18628
10	(child* abuse or child* neglect or battered child* or parent-infant traumatic stress syndrome or shaken baby syndrome or shaken impact syndrome or whiplash-shake injury syndrome).ti,ab,kf.	21078
11	forensic*.ti,ab,kf.	84417
12	or/1-11	200622
13	preschool child/	722419
14	exp infant/	1356789
15	pediatrics/	104343
16	(child* or baby or babies or newborn* or new-born* or neonat* or neo-nat* or preterm* or pre-term* or prematur* or pre-matur* or infant* or toddler* or preschool* or pre-school* or kid or kids or boy* or girl* or pediatric* or paediatric* or under 5* or under five* or under two* or under 2* or under one* or under 1* or shaken impact syndrome or whiplash-shake injury syndrome or SBS).ti,ab,kf.	3983751
17	or/13-16	4503518
18	exp computer assisted diagnosis/ 1541825
19	exp "imaging and display"/ 2376007
20	(image* or imaging or MRI or CT or computed tomography or neuroimaging or radiolog* or radiograph* or bone survey* or skeletal survey* or bone scintigraphy or skeletal survey or virt?ops*).ti,ab,kf.	3586497
21	or/18-20	4705688
22	exp autopsy/ 220468
23	(autop* or preautop* or necrops* or necroscop* or obducti* or postmort* or post mort* or virt?ops*).ti,ab,kf.	394551
24	or/22-23	472626
25	12 and 17 and 21 and 24	593

**CINAHL Plus (EBSCOhost)**
**Search run: 15/07/2024**

(Expanders: Apply equivalent subjects
Search modes: proximity)

S1	(MH "Child Abuse+")	(25,000)
S2	(MH "Shaken Baby Syndrome")	(531)
S3	(MH "Forensic Medicine")	(3,988)
S4	TI ( (AHT or NAI or NAHI or IHT or SBS) ) OR AB ( (AHT or NAI or NAHI or IHT or SBS) )
(1,718)
S5	TI ( (abus* N4 (physical or suspect* or child* or infant* or injur* or "head trauma")) ) OR AB ( (abus* N4 (physical or suspect* or child* or infant* or injur* or "head trauma")) )	(19,095)
S6	TI ( ((inflicted or nonaccidental or "non-accidental") N2 (injur* or trauma or fracture*)) ) OR AB ( ((inflicted or nonaccidental or "non-accidental") N2 (injur* or trauma or fracture*)) ) (1,351)

S7 TI ( (maltreat* or "physical violence" or "physical punishment" or "short fall") ) OR AB ( (maltreat* or "physical violence" or "physical punishment" or "short fall") ) (8,846)
S8 TI ( ("child* abuse" or "child* neglect" or "battered child*" or "parent-infant traumatic stress syndrome" or "shaken baby syndrome" or "shaken impact syndrome" or "whiplash-shake injury syndrome") ) OR AB ( ("child* abuse" or "child* neglect" or "battered child*" or "parent-infant traumatic stress syndrome" or "shaken baby syndrome" or "shaken impact syndrome" or "whiplash-shake injury syndrome") ) (7,125)
S9 TI forensic* OR AB forensic* (11,843)
S10 S1 OR S2 OR S3 OR S4 OR S5 OR S6 OR S7 OR S8 OR S9 (51,892)
S11 (MH "Child, Preschool") OR (MH "Infant+") (410,024)
S12 (MH "Pediatrics") (21,364)
S13 TI ( (child* or baby or babies or newborn* or "new-born*" or neonat* or "neo-nat*" or preterm* or "pre-term*" or prematur* or "pre-matur*" or infant* or toddler* or preschool* or "pre-school*" or kid or kids or boy* or girl* or pediatric* or paediatric* or "under 5*" or "under five*" or "under two*" or "under 2*" or "under one*" or "under 1*" or "shaken impact syndrome" or "whiplash-shake injury syndrome" or SBS) ) OR AB ( (child* or baby or babies or newborn* or "new-born*" or neonat* or "neo-nat*" or preterm* or "pre-term*" or prematur* or "pre-matur*" or infant* or toddler* or preschool* or "pre-school*" or kid or kids or boy* or girl* or pediatric* or paediatric* or "under 5*" or "under five*" or "under two*" or "under 2*" or "under one*" or "under 1*" or "shaken impact syndrome" or "whiplash-shake injury syndrome" or SBS) ) (922,163)
S14 S11 OR S12 OR S13 (1,029,127)
S15 (MH "Diagnostic Imaging+") (515,100)
S16 TI ( (image* or imaging or MRI or "CT" or "computed tomography" or neuroimaging or radiolog* or radiograph* or "bone survey*" or "skeletal survey*" or "bone scintigraphy" or virt#ops*) ) OR AB ( (image* or imaging or MRI or "CT" or "computed tomography" or neuroimaging or radiolog* or radiograph* or "bone survey*" or "skeletal survey*" or "bone scintigraphy" or virt#ops*) ) (481,640)
S17 S15 OR S16 (757,452)
S18 (MH "Autopsy+") (6,000)
S19 TI ( (autop* or preautop* or necrops* or necroscop* or obducti* or postmort* or post mort* or virt#ops*) ) OR AB ( (autop* or preautop* or necrops* or necroscop* or obducti* or postmort* or post mort* or virt#ops*) ) (27,579)
S20 S18 OR S19 (29,185)
S21 S10 AND S14 AND S17 AND S20 (105)

**Web of Science Core Collection**
**Search run: 16/07/2024**

1 (AHT or NAI or NAHI or IHT or SBS) (Topic) or (abus* NEAR/4 (physical or suspect* or child* or infant* or injur* or "head trauma")) (Topic) or ((inflicted or nonaccidental or "non-accidental") NEAR/2 (injur* or trauma or fracture*)) (Topic) or (maltreat* or "physical violence" or "physical punishment" or "short fall") (Topic) or ("child* abuse" or "child* neglect" or "battered child*" or "parent-infant traumatic stress syndrome" or "shaken baby syndrome" or "shaken

impact syndrome" or "whiplash-shake injury syndrome") (Topic) or forensic* (Topic) 210,863

2 (child* or baby or babies or newborn* or "new-born*" or neonat* or "neo-nat*" or preterm* or "pre-term*" or prematur* or "pre-matur*" or infant* or toddler* or preschool* or "pre-school*" or kid or kids or boy* or girl* or pediatric* or paediatric* or "under 5*" or "under five*" or "under two*" or "under 2*" or "under one*" or "under 1*" or "shaken impact syndrome" or "whiplash-shake injury syndrome" or SBS) (Topic) 4,031,310

3 TS=((image* or imaging or MRI or CT or "computed tomography" or neuroimaging or radiolog* or radiograph* or "bone survey*" or "skeletal survey*" or "bone scintigraphy" or virt$ops*)) 4,544,157

4 TS=((autop* or preautop* or necrops* or necroscop* or obducti* or postmort* or "post mort*" or virt$ops*)) 326,875

5 #1 AND #2 AND #3 AND #4 359
